# A novel cultivation strategy to recover NK cell cytotoxicity

**DOI:** 10.3389/fbioe.2026.1797129

**Published:** 2026-04-02

**Authors:** Valentin von Werz, Aleksander Szarzynski, Matthias Hadrbolec, Gregor Mattert, Sara Zigon-Branc, Bence Kozma, Werner Dammermann, Oliver Spadiut

**Affiliations:** 1 Research Area Biochemical Engineering, Institute of Chemical, Environmental and Bioscience Engineering, TU Wien, Vienna, Austria; 2 Center for Translational Medicine Germany, University Hospital Brandenburg, Brandenburg Medical School Theodor Fontane, Brandenburg an der Havel, Germany; 3 Faculty of Health Sciences Brandenburg, Brandenburg an der Havel, Germany; 4 Department of Gastroenterology, Diabetology and Hepatology, Center for Internal Medicine II, University Hospital Brandenburg, Brandenburg Medical School Theodor Fontane, Brandenburg an der Havel, Germany

**Keywords:** bioreactor, critical quality attribute, cytotoxicity, cytotoxicity recovery, design of experiments, lactate, NK cells, NK-92 cell line

## Abstract

Natural killer cells are emerging as promising “off-the-shelf” effectors for cancer immunotherapy, yet expansion of the NK-92 cell line in batch cultivation leads to rapid loss of cytotoxicity concomitant with lactate accumulation. In this study we developed and validated a two-phase manufacturing strategy that decouples cell proliferation from functional recovery in order to obtain an improved final product potency. Our 8-day kinetic survey determined declines in viability, metabolite profiles and cytotoxicity during static batch expansion. Guided by these data, in a 32-run full-factorial design-of-experiments approach we varied fresh cultivation medium proportion, temperature, dissolved oxygen, and recovery duration; partial least squares modeling identified fresh-medium ratio and recovery time as the primary drivers of cytotoxicity restoration. Identified optimal conditions (90% fresh medium, 37.2 °C, 3.7 days recovery) recovered cytotoxicity and maximized cytotoxic capacity. These setpoints were then translated to a 2 L stirred-tank bioreactor, where a fed-batch expansion under controlled pH and lactate levels produced 2.0 × 10^9^ cells, followed by recovery that achieved 43% ± 8% cytotoxicity. This scalable, two-phase paradigm minimizes medium usage and obviates continuous perfusion, offering a potential workflow to increase NK-92 potency and a base for manufacturing high-quality advanced therapy medicinal products.

## Graphical Abstract

**Figure:**
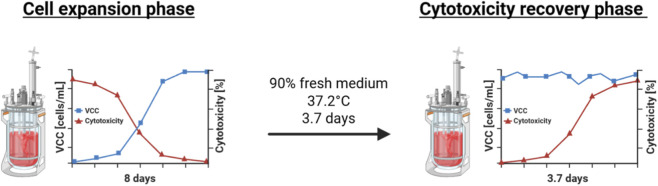

## 1 Introduction

Cellular immunotherapies have revolutionized the treatment landscape against cancer by offering targeted, effective solutions for previously incurable diseases (Qin et al., 2025). The clinical success of chimeric antigen receptor-modified T (CAR-T) cells, particularly against B-cell leukemia and lymphoma, underscores the transformative power of these new modalities (Zhong et al., 2024). Yet, CAR-T therapies pose considerable challenges: patient-specific manufacturing is labor-intensive and time-critical, severe toxicities such as cytokine-release syndrome and immune cell associated neurotoxicity syndrome demand intensive-care resources, and efficacy in solid tumors remains limited by the immunosuppressive tumor microenvironment (June et al., 2018; Westin and Sehn, 2022). These shortcomings have catalyzed the search for alternative, “off-the-shelf” cell types with complementary biology. Natural killer (NK) cells combine innate immune recognition with potent cytotoxicity and minimal risk of graft-versus-host disease, making them suited for allogeneic use (Rebuffet et al., 2024). Unlike T cells, NK cells survey for “missing-self” signals and stress ligands rather than a single defined antigen, enabling them to target a broad range of hematologic and solid tumors (Look et al., 2025). Engineering strategies - including CAR expression, armored cytokine support, and checkpoint blockade - are rapidly expanding NK functionality, and a growing number of clinical trials has demonstrated favorable safety profiles (Shah et al., 2015; McKenna et al., 2012; Dolstra et al., 2017; Williams et al., 2017; Boyiadzis et al., 2017).

Within this landscape, the NK-92 cell line occupies a unique niche. Derived from a patient with non-Hodgkin’s lymphoma, NK-92 cells exhibit homogeneous killer-immunoglobulin-like receptor negativity, high cytotoxicity, and amenability to large-scale expansion and genetic modification (Klingemann and Miyagawa, 1996; Lamers-Kok et al., 2022; Kotzur et al., 2022; Chrobok et al., 2019). Clinical phase I/II trials have verified the post-irradiation safety of NK-92 cells (Williams et al., 2017; Arai et al., 2008; Tonn et al., 2001), firmly positioning this cell line as a well-characterized platform for both unmodified and CAR-engineered therapeutics (Wlodarczyk and Pyrzynska, 2022). Nevertheless, translating laboratory protocols into cGMP-compliant, industrial manufacturing remains non-trivial: high cell doses (≥10^9^ cells per patient) must be generated rapidly while also preserving their cytotoxic potency—arguably the most critical quality attribute (CQA) for therapeutical efficacy (Motallebnejad et al., 2023).

In bioprocessing, product quality is defined by CQAs as physical, chemical, biological, or microbiological properties or characteristics that should be within an appropriate limit, range, or distribution to ensure the desired product quality (U.S. Department of Health and Human Services, Food and Drug Administration, 2006; ICH et al., 2009). To achieve these CQAs and meet the specifications, it is essential to identify the critical process parameters (CPPs). CPPs are process parameters whose variability has an impact on a CQA and therefore should be monitored or controlled to ensure the process produces the desired quality (U.S. Department of Health and Human Services, Food and Drug Administration, 2006). In recent years, several contributions have introduced and suggested specified release tests for NK cell CQAs, similar to those used in the manufacturing of other cell therapies (Jahan et al., 2024; von Werz et al., 2025; Deng et al., 2025; Shap et al., 2025; Tang et al., 2018). However, the CQAs and CPPs have not yet been fully established, which make it challenging to ensure that the final product consistently meets the required standards for safety, identity, purity, content/strength and potency (von Werz et al., 2025; von Werz et al., 2024; Amr Eissa, 2021).

Bioprocess engineers ensure CQAs by optimizing CPPs (such as nutrient levels, cultivation pH, temperature, dissolved oxygen tension, and metabolite load) and by selecting appropriate cultivation modes to prevent nutrient depletion and/or metabolite accumulation, whose effects on NK cytotoxicity remain poorly determined (Lapteva et al., 2014; Garcia-Aponte et al., 2021). Due to their simplicity and wide laboratory applicability, batch cultivations (i.e., cultivations where no nutrients are added to the vessel during cultivation) are still prevalent for NK-92 expansion, but also highly prone to progressive nutrient depletion and metabolite build-up (Motallebnejad et al., 2023). For example, lactate accumulation is a well-known growth inhibitor whose influence on NK cytotoxicity remains limited (Huang et al., 2023; Jin et al., 2025; Caslin et al., 2021; Li et al., 2022; Jedlicka et al., 2022). More sophisticated modes such as fed-batch (where nutrients are added systematically for an extended duration), perfusion (with continuous supply of fresh medium and removal of spent medium), or semi-continuous cultivation mitigate metabolic stress but require automated bioreactors, advanced sensors, and higher cultivation medium volumes which are increasing the cost of the overall cultivation process (Garcia-Aponte et al., 2021). Recent state-of-the-art solutions for NK cell expansion include gas-permeable static vessels (e.g., G-Rex by ScaleReady), closed platforms (e.g., CliniMACS Prodigy by Miltenyi Biotec), fixed-bed perfusion reactors (e.g., iCellis by Cytiva), or micro-bioreactors (e.g., Ambr250 by Sartorius) (Motallebnejad et al., 2023; Jahan et al., 2024; Garcia-Aponte et al., 2021). These technologies offer varying degrees of automation and closed processing, but at a higher expense. A definitive consensus on NK-92 manufacturing has yet to emerge, because current platforms differ greatly in throughput, footprint, regulatory complexity and, importantly, the overall cost of therapy.

Emerging evidence indicates that NK-92 cytotoxicity is highly dynamic (Aponte et al., 2023): peak effector function coincides with early-to mid-log-phase growth and deteriorates as cultivations approach the stationary phase (Garcia-Aponte et al., 2021). Previous studies implicate nutrients depletion and metabolite accumulation (e.g., glucose, glutamine, and pyruvate (Kern Coquillat et al., 2025), or ammonia and lactate (Huang et al., 2023; Kern Coquillat et al., 2025; Scott and Cleveland, 2016; Quinn et al., 2020)), altered redox balance (Quinn et al., 2020), shifts in pH (Cappellesso et al., 2024) or oxygen levels (Mirchandani et al., 2025) as contributing factors, yet systematic, time-resolved analyses are scarce (Furuke et al., 1999).

Importantly, immune cell function is frequently lost in lactate-rich, hypoxic tumor microenvironments (TME) often exceeding 20 mM lactate (Jin et al., 2025; Wang et al., 2023; Du et al., 2021; Zhou et al., 2023; Kim et al., 2025; Brand et al., 2016; Husain et al., 2013). Beyond classical acetylation, recently described post-translational modifications, such as protein and histone lactylation, underscore the clinical relevance of metabolite-driven NK dysfunction (Jin et al., 2025; Zhang et al., 2019; Lopez Krol et al., 2022). In addition, oxygen availability critically shapes NK cell metabolism and effector function, as hypoxic conditions can impair cytotoxicity, alter cytokine secretion profiles, and induce metabolic reprogramming toward glycolysis (Retamal et al., 2025; Chang et al., 2025). Fluctuations in dissolved oxygen during large scale cultivation may therefore directly influence product potency and represent an underappreciated CPP. Nevertheless, manufacturing protocols still mostly rely on conservative harvest windows or endpoint cytotoxicity assays that capture only snapshots of cell functionality, potentially leading to missed optimal harvest windows and an increased risk of a batch failure. A mechanistic, time-resolved understanding of cytotoxicity dynamics is therefore needed and indispensable—not only to secure future consistent therapeutic potency in patients but also to ensure scalable and therapeutically more efficient off-the-shelf NK-92 production.

In this study we hypothesized that by separating the cell proliferation phase from the functional recovery phase of NK-92 cells, the optimized expansion modality would grant greater manufacturing flexibility, reduce medium consumption and ultimately secure the product potency. In brief, we conducted an 8-day kinetic survey to quantify cytotoxicity, viable cell count, viability and extracellular metabolites, establishing a baseline trajectory of a functional decline. Guided by these data, in a 32-run design-of-experiments (DoE) we varied the factors proportion of fresh cultivation medium, cultivation temperature, dissolved oxygen and recovery duration, and modelled their main and interaction effects on cytotoxicity and on cytotoxic capacity—a novel key performance indicator metric defined as the product of cytotoxicity and total viable cell count, enabling a direct comparison of the overall product potency under standardized conditions (von Werz et al., 2026). The resulting optimal set-points were validated in a 2-L stirred-tank bioreactor (STR) with controlled pH and lactate-based feeding.

## 2 Materials and methods

### 2.1 Static cultivation

NK-92 cells (ACC 488, Leibniz Institute DSMZ-German Collection of Microorganisms and Cell Cultures GmbH, Braunschweig, Germany) were cultivated at standard cultivation conditions (37 °C and 5% CO_2_ in a humidified incubator), in a minimal essential medium alpha modification (aMEM; Gibco, Paisley, United Kingdom) supplemented to a final concentration of 1.15 mM L-Arginine (Carl Roth, Karlsruhe, Germany), 2.31 mM L-Glutamine (Carl Roth, Karlsruhe, Germany), 0.286 mM L-Serine (Thermo Fisher Scientific, Waltham, MA, United States), 0.194 mM i-inositol (Sigma-Aldrich, St. Louis, MO, United States), 23.2 mM D-Glucose (Carl Roth, Karlsruhe, Germany), 24 µM beta-mercaptoethanol (Sigma-Aldrich, St. Louis, MO, United States), Insulin-Transferrin-Selenium (Gibco, Grand Island, NY, United States) 17.21 µM, 0.69 µM and 0.39 µM, respectively, 2.2 g/L Sodium bicarbonate (Sigma-Aldrich, St. Louis, MO, United States), 500 IU/mL recombinant premium grade interleukin-2 (IL-2; Miltenyi Biotech, Bergisch Gladbach, Germany) and 10% type AB, off-the-clot human serum (Pan Biotech, Aidenbach, Germany). A batch cultivation was seeded in T-175 flasks (Starlab, Brussels, Belgium) at a concentration of 2.5 × 10^5^ cells/mL and was supplemented with IL-2 every second day without media exchange or addition.

To assess the cytotoxicity of the NK-92 cells, K-562-GFP (K562) cells (CCL-243-GFP, American Type Culture Collection, Manassas, VA, United States) were maintained at standard cultivation conditions in a Roswell Park Memorial Institute 1640 medium (RPMI; Gibco, Grand Island, NY, United States) containing 5% heat-inactivated fetal bovine serum (FBS, Gibco, Thermo Fisher Scientific, Waltham, MA, United States). Regular subculture of K562 cells was done at a seeding concentration of 1 × 10^6^ cells/mL.

### 2.2 Cytotoxicity recovery

On cultivation day 7, NK-92 cytotoxicity against K562 cells was assessed, and the cytotoxicity recovery was initiated by reseeding the cells at a concentration of 2.5 × 10^5^ cells/mL in a total of 8 mL per cultivation.

The experimental plan of the cytotoxicity recovery phase was planned according to the DoE principle, using the software MODDE 13.0.2 (Sartorius, Göttingen, Germany). A four-factor, full-factorial design was chosen, with two levels for the factors of temperature and oxygen level, three levels for the factor cultivation medium composition, and four levels for the factor cultivation duration, defining the experimental design space (Table 1 and Supplementary Table S1). At 0% fresh medium setpoint, the spent cultivation medium was used that was harvested as supernatant on day 7 from the batch cultivation by removing all cells and debris with microfiltration (0.2 µM PES membrane (Starlab, Brussels, Belgium)). The 50% setpoint consisted of a 1:1 mixture of spent and fresh cultivation medium. The analyzed parameters in the DoE, also called responses, were cytotoxicity and cytotoxic capacity. The design space was divided into four sets of runs, where each run was represented by a single experimental vessel, based on the four different cultivation duration levels, placed into different CO_2_ incubators, set to the respective oxygen and temperature setpoints (Table 1 and Supplementary Table S1). To prevent negative effects of repeated cell disturbance, one cultivation vessel was seeded for each harvest timepoint defined by the recovery duration to ensure homogeneous cultivation conditions for all vessels and the entire recovery phase. For each set of runs, two independent center points were included in triplicates to assess the biological variance, resulting in a total of 64 runs (Supplementary Table S1).

**TABLE 1:** Summary of the factors and the respective levels for the full factorial DoE. Two independent center-points (in triplicates per level) of the factor cultivation duration were included. A graphical representation of the design space is shown in Supplementary Figure S2A.

Factor	Unit	Lowest level	Lower level	Higher level	Highest level
Fresh medium ratio	%	0	50		100
Oxygen concentration	%	10	—	—	21
Temperature	°C	37	—	—	39
Cultivation duration	days	1	2	3	4

### 2.3 Stirred-tank bioreactor cultivation

Bioreactor cultivations were conducted in a 2-L working volume UniVessel glass vessels (Sartorius, Göttingen, Germany) controlled by Labfors 5 (Infors HT, Bottmingen, Switzerland) controllers. Each vessel was equipped with two, 30° 3-blade pitch-blade impellers, a microsparger for aeration (both Sartorius, Göttingen, Germany), pH and optical dissolved oxygen (dO) sensors (both Hamilton, Bonaduz, Switzerland). The cultivations were heated with external heating mats and the temperature monitoring was performed using the built-in Pt100 probe. The inoculum was expanded within 250 mL shake flasks (Corning, Glendale, AZ, United States) positioned on an orbital shaker under standard cultivation conditions at 120 rpm. During pre-culture expansion NK-92 cells were seeded at 2.5 × 10^5^ cells/mL, IL-2 was supplemented every second day, and medium was exchanged every 2–4 days. The inoculum in the bioreactor was also seeded at 2.5 × 10^5^ NK-92 cells/mL in a 600 mL starting volume (80 rpm, 37 °C) with IL-2 supplementation every second day. A total gas flow rate of 12 mL/min (0.02 vessel volumes per minute) was achieved by a constant pressurized air flow of 2 mL/min, varying pure oxygen flow to maintain a dO level of 60%, varying pure CO_2_ flow to sustain 5% CO_2_ level in the off-gas and varying pure nitrogen flow to reach the total gas flow rate, all controlled by mass flow controllers. The off-gas left the reactor through a chilled off-gas cooler and its O_2_, CO_2_ and humidity content were analyzed by a BlueInOne Cell gas analyzer (BlueSense, Herten, Germany). The pH was maintained at 7.40 (±0.05) with the addition of 0.5 M NaHCO_3_, delivered through the integrated pump system of the controller. The batch phase was terminated, when the lactate concentration exceeded a threshold of 12 mM by feeding a total of 1000 mL fresh medium in an exponential feed regime (Li et al., 2015), using PreciFlow (Lambda Instruments, Baar, Switzerland) peristaltic pumps, with the goal to maintain a lactate concentration below this threshold. The initial feed rate was determined based on the lactate concentration (Equations 1 and 2) and an exponential feed rate was set according to the growth rate of the cells (Equations 3 and 4). The cultivation was maintained after all feed was added to the cultivation, until no further growth was observed (Costariol et al., 2019). After the cultivation ended, cells were separated from the cultivation medium by centrifugation at 300 × *g* for 5 min at RT, and the cytotoxicity recovery phase was initiated by reseeding a fraction (6%) of the cells in 90% (v/v) of fresh medium at a seeding density of 2.5 × 10^5^ cells/mL in a total volume of 600 mL. For the recovery phase, the bioreactor was maintained at 37.2 °C, 5% CO_2_ in the off-gas, 52.86% dO, and a pH of 7.4, with a stirring speed of 80 rpm and a total gas flow rate of 12 mL/min, as the predicted optimum of our DoE analysis in T-Flasks from this study. Throughout the cultivation and the cytotoxicity recovery process, all PPs were monitored and recorded by the Lucullus process information management system (Securecell, Urdorf, Switzerland).

$$F_{t_{n+1}}=\left(\frac{c_{Lactate\;t_{n+1}}\cdot {V_{reactor}}_{t_{n}}}{c_{Lactate}^{Setpoint}}\right)-{V_{reactor}}_{t_{n}}$$ (1)

Equation 1 Feedrate ( $F_{t_{n+1}}$ ) based on the lactate concentration at time point $t_{n+1}$ ( $\left.c_{Lactate\;t_{n+1}}\right)$ the desired lactate setpoint ( $c_{Lactate}^{Setpoint}$ ) and the current reactor volume ( ${V_{reactor}}_{t_{n}}$ ).

$$c_{Lactate\;t_{n+1}}=c_{Lactate\;t_{n}}+q_{Lactate\;t_{n}}\cdot {VCC}_{t_{n}}\cdot \;\Delta t$$ (2)

Equation 2 Calculation of the lactate concentration ( $\left.c_{Lactate\;t_{n+1}}\right)$ at the time point $t_{n+1}$ determined as the sum of the current lactate ( $c_{Lactate\;t_{n}}$ ), and the product of the specific lactate production rate ( $q_{Lactate\;t_{n}}$ ) at the time point $t_{n}$ , total viable cell count at the time point $t_{n}$ ( $\left.{VCC}_{t_{n}}\right)$ and the time difference Δt between t_n_ and t_n+1_.

$$µ=\frac{\ln\left(N_{t}/N_{t0}\right)}{\left(T_{1}-T_{0}\right)}$$ (3)

Equation 3 Calculation of the specific growth rate µ where *N*_*t*_ is the total viable cell count at the selected time point, and *N*_t0_ is the initial viable cell count. *T*_1_ is the current time point, and *T*_0_ is the time point of seeding.

$$F\left(t\right)=F_{0}\cdot e^{\mu *t}$$ (4)

Equation 4 Calculation of the exponential feed rate based on the initial feeding volume (*F*_0_) multiplied by the exponent (*e*) of the specific growth rate (*µ*) and time (*t*).

### 2.4 Process analytics

#### 2.4.1 Cell counting and viability

Cell count and viability were measured by flow cytometry on a CytoFLEX S (Beckman Coulter, Brea, CA, United States). 150 μL of cell suspension was mixed with 50 µL of cell counting beads (Invitrogen, Thermo Fisher Scientific, Waltham, MA, United States) and 50 µL of 5 μg/mL 7-AAD (Invitrogen, Thermo Fisher Scientific, Waltham, MA, United States) fluorescent stain for live-dead cell discrimination. The viable cell concentration was calculated as described in Equation 5.

$$Viable\;cell\;count\;\left[\frac{cells}{µL}\right]=\frac{\left({7AAD}^{-}\text{event}\;\text{count}\;x\;counting\;bead\;volume\right)}{\left(counting\;bead\;count\;x\;cell\;volume\right)}\;x\;counting\;bead\;concentration$$ (5)

Equation 5 Formula for the calculation of the absolute cell count as instructed by the manufacturer of the counting beads. Cell count and bead count are determined by the respective event count in the gates by flow cytometry.

Size calibration beads for flow cytometry (Invitrogen, Thermo Fisher Scientific, Waltham, MA, United States) were used to perform a size calibration that enabled the gating of particles based on the beads’ reference size. This calibration step minimized background signals in live-dead cell discrimination, which primarily resulted from protein aggregation in the medium, by excluding particles smaller than 8 μm, which is less than half the size of a NK-92 cell which is 10–12 µm (Klingemann, 2023). Afterwards, viable cells were gated with the side-scatter signal, including only 7-AAD^-^ events. The bead count was gated according to the manufacturer’s protocol in a fluorescent channel (660 ± 10 nm detector) unaffected by other stains used in the analysis.

#### 2.4.2 Cell growth

The cell growth was determined by the calculation of the population doublings (Equation 6) within a defined duration, based on the equation used by Hood et al. (Hood et al., 2024).

$${PD}_{n}=\frac{1}{\ln\;2}*\ln\frac{C_{x,n}}{C_{x,n-1}}$$ (6)

Equation 6 Population doublings were calculated as *PD_n_* where *C_x,n_* was the viable cell concentration at a given timepoint and *C_x,n-_*_1_ was the viable cell concentration on the previous day. For the calculation of cumulative population doublings (*cPD*), *C_x,n-_*_1_ is replaced with *C_x,n_*_0_.

#### 2.4.3 Cytotoxicity and cytotoxic capacity determination

Cytotoxicity of NK-92 cells was assessed by co-culturing them with K562 cells for a duration of 4 h, at an effector-to-target (E:T) ratio of 1:1. Both cell types were seeded in triplicates at a concentration of 1 × 10^5^ cells/well in 96-well V-bottom plates (Thermo Fisher Scientific, Waltham, MA, United States) in 200 µL aMEM medium devoid of serum and IL-2. Additionally, a negative control of both cell types containing the same cell amount (2 × 10^5^ cells/well) was seeded to assess spontaneous death. After 4 h, the cells were collected by centrifugation (300 x *g*, 5 min), washed in a FACS buffer (containing PBS (Gibco, Paisley, United Kingdom) + 1% bovine serum albumin (Carl Roth, Karlsruhe, Germany) + 50 µM NaN_3_ (Thermo Fisher Scientific, Waltham, MA, United States)), collected again (300 x *g*, 5 min), then stained with Annexin-V (BD Biosciences, Franklin Lakes, NJ, United States) for apoptosis. The reaction was carried out by adding 100 µL annexin binding buffer (ABB; Invitrogen, Thermo Fisher Scientific, Waltham, MA, United States) to each well (which contained 0.625 µL Annexin-V) and incubated for 15 min at RT in the dark. Afterwards 150 µL ABB was added and cells were collected by centrifugation. After removal of the supernatant cells were resuspended in 250 µL 7-AAD in ABB to a final concentration of 1 μg/mL for the second staining step, where Annexin^+^ 7-AAD^-^ events indicated early apoptotic cells, Annexin^+^ 7-AAD^+^ events indicated late apoptotic cells, and Annexin^−^ 7-AAD^+^ events indicated necrotic cells. After the second staining, the cells were immediately analyzed by flow cytometry. For each measurement, a minimum of 10,000 events were recorded in the gate of live K562 or NK-92 cells, respectively.

Cytotoxicity was calculated by quantifying the loss of viable target cells in the co-culture with NK-92 cells, compared to the negative controls as recommended by the distributor of the target cells (Equation 7) (Penney McWilliams-Koeppen, 2022). Therefore, the count of early apoptotic, late apoptotic, and necrotic K562 cells were used from the co-culture sample and the negative control sample.

$$Cytotoxicity\;\left[\%\right]=\frac{\left(100\%-live\;K562\;\left[\%\right]\right)-\left(100\%-live\;K562_{control}\left[\%\right]\right)}{live\;K562_{control}\left[\%\right]}x100$$ (7)

Equation 7 Formula for the determination of absolute cytotoxicity from flow cytometry measurements where “live K562” are 7-AAD and Annexin-V double-negative effector events in co-culture wells, and “live K562_control_” are the 7-AAD and Annexin-V double-negative effector cells in the control wells.

In addition, the cytotoxic capacity was calculated to describe the potential of the whole culture to kill a target cell within 4 h of co-culture (Equation 8).

$$Cytotoxic\;capacity\;\left[\text{cells}\right]=\text{cytotoxicity }\left[\%\right]x\text{ total viable cells }\left[\text{cells}\right]$$ (8)

Equation 8 Formula for the calculation of the cytotoxic capacity of the cultivation, where the cytotoxicity is multiplied by the total viable cell count of the cultivation. The result is a number of cells that have the capacity to eliminate the same number of target cell within 4 hours of incubation.

To compare the overall productivity between media formulations and process modes, space-time yield (STY) was calculated as already reported for other products (Bausch et al., 2019) (Equation 9).

$$STY\;\left[cytotoxic\;cells\right]=\frac{\left(Cytotoxic\;capacity\;\left[cells\right]\;T_{1}-Cytotoxic\;capacity\;\left[cells\right]\;T_{0}\right)\;}{V\;\left[L\right]*t\;\left[hours\right]}$$ (9)

Equation 9 Calculation of the space-time yield (STY), where the cytotoxic capacity at timepoint *T*_0_ is the timepoint of inoculation and timepoint *T*_1_ is the timepoint of harvest, *V* is the total volume used for expansion and t is the process duration.

#### 2.4.4 Medium analysis

From the spent medium, the concentrations of glucose, lactate, glutamine, and ammonia were determined by a CEDEX BioHT analyzer (Roche, Basel, Switzerland). The medium pH was also measured, but only after re-equilibrating the spent medium in a 5% CO_2_ incubator environment for at least 1 h, in order to minimize pH shifts caused by the degassing of CO_2_. Afterwards, the pH was measured immediately after removing the sample from the incubator, using a freshly calibrated pH sensor (Xylem Analytics, San Diego, United States).

#### 2.4.5 Cell surface marker analysis

On day 3 of cytotoxicity recovery in small-scale DoE experiments, 1 × 10^6^ cells were fixed for surface marker analysis according to the manufacturers protocol using Cytofix (BD, Franklin Lakes, NJ, United States). In brief, cells were collected by centrifugation at 300 × *g* for 5 min at RT and washed two times in FACS buffer. Next, the cell pellet was fixed in Cytofix fixation buffer for 15 min at 4 °C, washed in FACS buffer and then frozen at −80 °C in freezing medium (FBS + 10% Dimethylsulfoxid (Sigma-Aldrich, St. Louis, MO, United States)). Flow cytometry analysis was performed on a SymphonyA3 flow cytometer (BD, Franklin Lakes, NJ, United States). After thawing, the cells were incubated within a Human BD Fc Block (BD, Franklin Lakes, NJ, United States) for 10 min. Antibody staining was performed using the specified volumes of each antibody (Supplementary Table S2) for 30 min at RT in the dark, separating all analyzed markers into two panels. Gates for all markers were defined using fluorescence minus one. Binding specificity was confirmed using isotype controls. The data was analyzed and visualized using FlowJo software (version 10.10.0, BD, Franklin Lakes, NJ, United States). For each cultivation condition (fresh, mixed, and spent cultivation medium), samples were concatenated and stacked histograms generated.

### 2.5 Data analysis

Time-resolved analysis of the cultivation data from batch expansion was performed by combining three independent biological replicates and presenting their standard deviation. Data obtained by the recovery experiment was shown with the standard deviation of all runs that used the same medium composition. Raw results of cytotoxicity and cytotoxic capacity without preprocessing were used for the generation of the PLS model using MODDE 13.0.2 to describe the data and identify its significant factors. An automatic factor selection was performed by MODDE to exclude factors insignificant to model predictions for each response. Statistical significance of model coefficients was evaluated at a 95% confidence level. The model performance was assessed by its descriptive power *(R* ^2^ *)* and its predictive power *(Q* ^2^ *)*, both of which should be maximized. *Q* ^2^ was calculated using leave one out based cross validation. A difference of less than 0.2 between *R* ^2^ and *Q* ^2^ indicates a robust model (Hood et al., 2024). After exclusion of insignificant factors, a second PLS model, with one component/latent variable for cytotoxicity and one component/latent variable for cytotoxic capacity (Supplementary Table S3), was fitted to the data to predict the response outcome within the whole design space. No data transformation and outlier exclusion was performed for the model generations. The theoretical combined optimum for both responses was identified in MODDE by finding the theoretical conditions that maximized both cytotoxicity and cytotoxic capacity in the model predictions. The data obtained from the verification of the optimized setpoints in a stirred-tank bioreactor was shown with standard deviations of three technical replicates. Statistical analysis was performed in GraphPad Prism (version 10.4.0 for Windows, GraphPad Software, Boston, MA, United States). A correlation analysis with a selection of PPs and cellular QAs was performed using Pearson correlation. The variance of the means was determined by one-way ANOVA with *post hoc* pairwise comparison using the Tukey-Kramer test. For comparison of two datasets, an ordinary t-test was chosen. The following levels of significance were assigned if the p-values where p ≥ 0.05 is not significant (ns), p < 0.05 is *, p < 0.01 is **, p < 0.001 is ***, and p < 0.0001 is ****. Graphical overview figures were created using Biorender.com (Biorender, Toronto, Canada). Bar and line charts were created with the GraphPad Prism software.

## 3 Results

### 3.1 Loss of cytotoxicity during expansion

**FIGURE 1:**
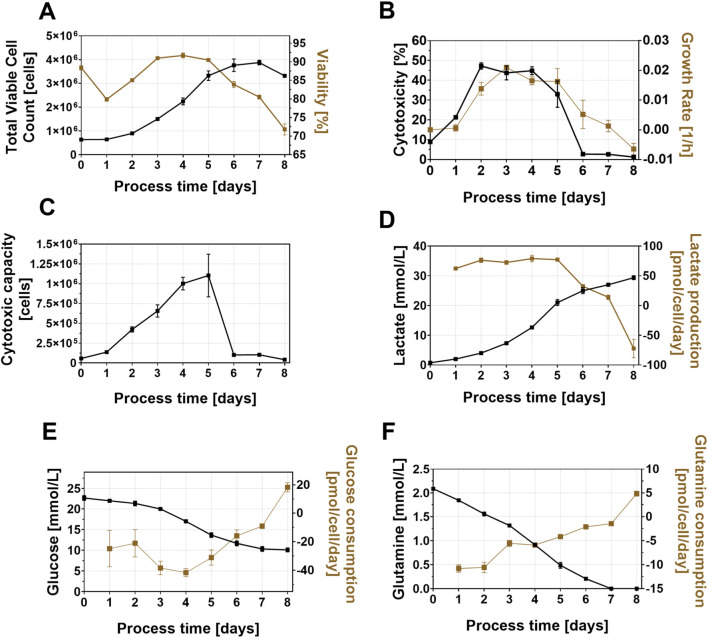
Total VCC, viability **(A)**, cytotoxicity, specific growth rate **(B)**, cytotoxic capacity **(C)**, lactate concentration and production rate **(D)**, glucose concentration and consumption rate **(E)**, glutamine concentration and consumption rate **(F)** of the NK-92 cell batch expansion process in static T-flasks. Error bars represent the standard deviation of three, independent biological replicates (n = 3).

Cultivating NK-92 cells in a batch process over 8 days resulted in a growth curve with distinct lag, exponential, plateau and decline phases (Figure 1A). Viability was maintained around 90% during exponential growth but dropped significantly in later phases. Cytotoxicity against K562 cells peaked early at 43.7% ± 2.92% on day 2, but decreased rapidly afterward, reaching a minimal of only 1.23% ± 0.08% by day 8 (Figure 1B). This decline also impacted on the cytotoxic capacity, which peaked on day 5 and sharply fell thereafter, emphasizing that high cell count alone is insufficient for an effective product without a maintained cytotoxic functionality (Figure 1C). Metabolically, nutrient consumption clearly correlated with growth, and the rapid decline in cytotoxicity indicated that the batch cultivation alone was unable to sustain a high functionality of the cells (Figures 1D–F). Due to this rapid loss of cytotoxicity, further process optimization steps—such as modifying nutrient supply or employing alternative process modes—are necessary to recover the cytotoxicity and ensure an effective therapeutic product. We next sought to understand whether a therapeutically relevant number of cells could be produced in a batch process mode while also enabling a recovery of the cytotoxicity before infusion.

### 3.2 Recovery of cytotoxicity after expansion

#### 3.2.1 Impact of cultivation parameters on cytotoxicity during recovery

**FIGURE 2:**
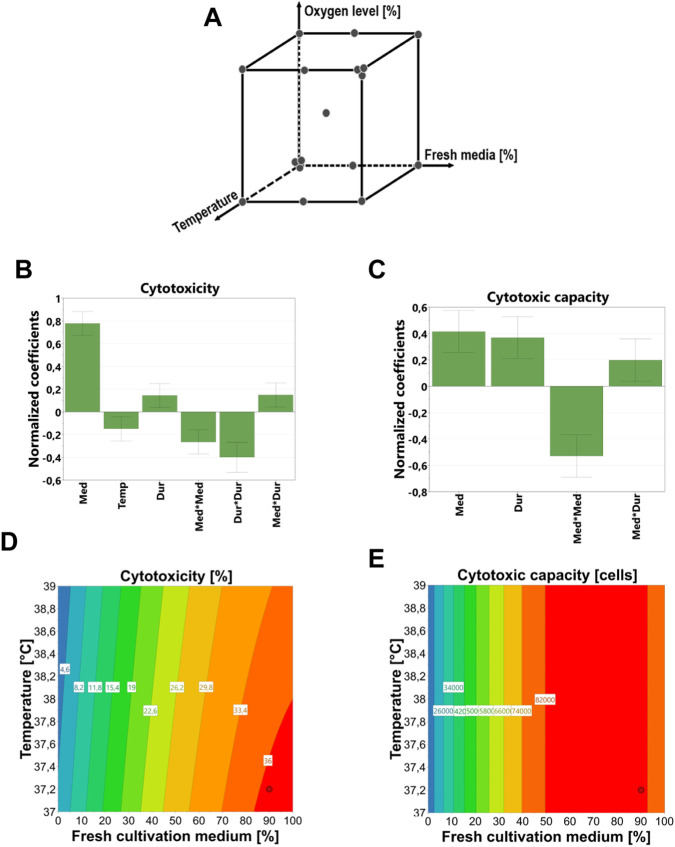
**(A)** Graphical overview of the design space used to determine the cytotoxicity recovery without an additional factor of recovery duration. Overview of significant factors used for the calculation of the PLS model for cytotoxicity **(B)** and cytotoxic capacity **(C)** after normalization of the coefficients. Only significant factors are displayed (different than zero) with a standard deviation smaller than the factor’s value - Med: medium composition, Temp: cultivation temperature Dur: recovery duration. **(D,E)** Contour plots illustrating PLS-based predictions of the optimal recovery setpoint, derived from the analysis of DoE responses for cytotoxicity and cytotoxic cells. The circles on both plots represent the combined optimum conditions that maximize cytotoxicity and cytotoxic capacity recovery. The factor recovery duration was set to the identified optimum of 3.7 days.

**FIGURE 3:**
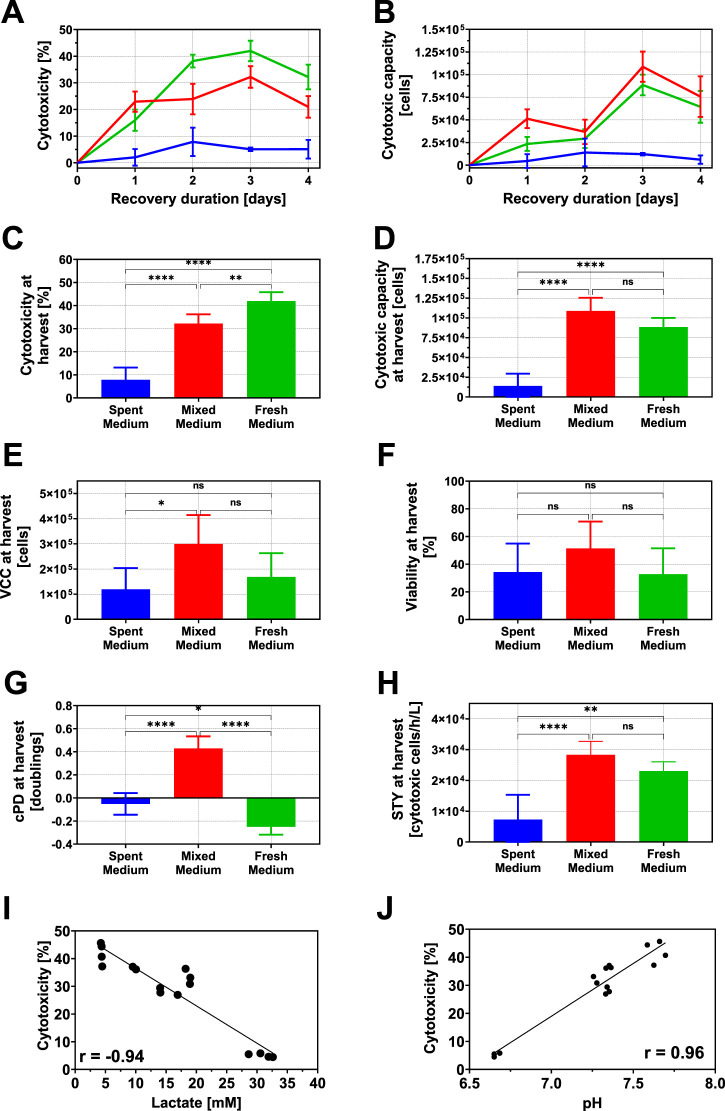
Time-resolved measurements of cytotoxicity **(A)** and cytotoxic capacity **(B)** during the cytotoxicity recovery process. Cytotoxicity **(C)**, cytotoxic capacity **(D)**, total viable cell count **(E)**, viability **(F)**, cumulative population doublings **(G)**, and STY **(H)** were compared between medium compositions on day 3 when cytotoxicity recovery reached its maximum, using one-way ANOVA, where p ≥ 0.05 is not significant (ns), p < 0.05 is *, p < 0.01 is **, p < 0.001 is ***, and p < 0.0001 is ****. Error bars represent standard deviations of all runs per medium composition (n = 4 for fresh medium (green), n = 8 for mixed medium (red) and n = 4 for spent medium (blue)). Correlation between cytotoxicity at day 3 of the recovery and lactate concentration **(I)** and pH **(J)** at day 0 of cytotoxicity recovery. Regression was calculated with simple linear regression and correlation coefficients were calculated with Pearson’s correlation.

Following the batch expansion phase, NK-92 cells were subjected to a cytotoxicity recovery process under varying conditions defined by a DoE design space (Figure 2A and Supplementary Table S1). PLS regression modeling identified the cultivation medium composition (i.e., proportion of fresh cultivation medium added) as the most important factor contributing to cytotoxicity and cytotoxic capacity recovery, followed by recovery duration (Figures 3A,B). The effect of temperature was detectable, but minor (Figures 2B,C), while oxygen level was not proven to be significant and was therefore excluded from the model. Due to the non-linear recovery pattern, both cultivation medium composition and recovery duration were modeled additionally with quadratic terms (Figures 2B,C).

Time-resolved analysis showed that cytotoxicity increased progressively with recovery time in both fresh and mixed cultivation medium conditions, reaching a maximum on day 3 before declining (Figure 3A). This peak was significantly higher in cells cultivated in fresh cultivation medium (42% ± 3.3%) compared to mixed (32.2% ± 3.8%) and spent medium (5.1% ± 0.6%) (Figure 3C). Cytotoxic capacity, calculated as the product of cytotoxicity and total viable cell count, showed a similar trajectory (Figure 3B). On day 3, cells recovered in mixed cultivation reached a total of 108,767 ± 15,671 cytotoxic cells, which was approximately 22.9% more than observed with the cells cultivated in fresh cultivation medium (88,534 ± 9,927 cells) (Figure 3D). In contrast, cells in spent medium peaked earlier (on day 2), yielding only 12,217 ± 1,240 cytotoxic cells (only 11.2% of the mixed medium), reflecting their suppressed cytotoxic function (Figure 3D).

Overall, the time course data confirmed that the cytotoxicity recovery process was highly successful yet transient, with cytotoxicity and cytotoxic capacity peaking at day 3 in fresh and mixed medium and at day 2 in spent medium, followed by a decline. This suggested a defined window for optimal harvest following recovery.

#### 3.2.2 Cell growth and viability during recovery

To assess whether cytotoxicity recovery was accompanied by cell proliferation, total viable cell count, viability, and cumulative population doublings (cPD) were determined (Supplementary Figure S1A,B and Figure 3G). At the harvest point, VCC was significantly higher in the mixed cultivation medium group compared to spent cultivation medium, while the fresh cultivation medium group showed a lower average VCC, though differences between mixed and fresh media were not statistically significant (Figure 3E). Cell viability measurements paralleled these results: cells in mixed and fresh cultivation medium reached a viability of 57.7% ± 3.4% and 41.0% ± 3.1%, respectively, whereas cells in spent medium conditions showed reduced viability (39.9% ± 9.5%) (Figure 3F).

Only cells in mixed cultivation medium showed modest proliferation (0.43 ± 0.1 doublings) during the cytotoxicity recovery phase (Figure 3G). In contrast, cells in fresh and spent cultivation media did not proliferate and instead showed slight cell loss. The regression analysis between growth and cytotoxicity furthermore confirmed that cytotoxicity recovery was largely independent of proliferation (Supplementary Figure S1C).

#### 3.2.3 Cell surface markers during cytotoxicity recovery

With the aim to better understand the success of cytotoxicity recovery, we analyzed cell surface markers across the four functional groups on day 3 of the recovery process (Supplementary Figure S2). Apart from minor increases in CD15s (Sialyl-Lewis^×^ ligand) and CXCR3 (CD183) in fresh and mixed cultivation media, no differences emerged. Lineage markers (CD3, CD14, CD19, CD45, CD56) and activating receptors—including CD16, DNAM-1 (CD226), NKp46 (CD335), NKp44 (CD336), NKp30 (CD337), NKG2D (CD314) and TRAIL (CD253)—showed comparable expression across all cultivation media. Exhaustion markers PD-1 (CD279), LAG-3 (CD223), TIM-3 (CD366) and TIGIT were similarly low across conditions, as well as adhesion and homing receptors (CD62L, CD11a, integrin α4 (CD49d), PSGL-1 (CD162), CCR7 (CD197), CX3CR1, and CXCR4 (CD184).

#### 3.2.4 Association with metabolic waste products

To explore possible causes for the variation in cytotoxicity recovery outcomes between different cultivation media compositions, correlations between cytotoxicity and initial concentrations of metabolic products and pH were analyzed (Figures 3I,J, and Supplementary Figure S1D). The analysis revealed that the majority of metabolic products did not influence cytotoxicity recovery. However, cytotoxicity on day 3 exhibited a strong negative correlation with lactate concentration at the start of recovery (r = −0.94), as well as a positive correlation with pH (r = 0.96), consistent with the acidic nature of lactate accumulation. Theses suggest that elevated lactate levels and acidic conditions in the cultivation medium were detrimental to the success of cytotoxic recovery and potentially responsible for the loss during expansion, underscoring the importance of metabolite clearance and pH restoration.

#### 3.2.5 Prediction of optimal recovery setpoint

The fitted PLS models for cytotoxicity and cytotoxic capacity demonstrated good predictive power, with *R* ^2^ = 0.842/*Q* ^2^ = 0.787 and *R* ^2^ = 0.624/*Q* ^2^ = 0.600, respectively (Figures 2D,E). Based on these models, the predicted optimal conditions for maximizing both cytotoxicity and cytotoxic capacity were identified as 3.7 days of recovery duration, 37.2 °C, and 90% fresh medium. Under these setpoints, the model predicted a cytotoxicity of 36.5% and a cytotoxic capacity of 83,671 cytotoxic cells.

### 3.3 Verification of cytotoxicity recovery in a stirred-tank bioreactor

#### 3.3.1 Expansion dynamics under controlled pH and lactate conditions

**FIGURE 4:**
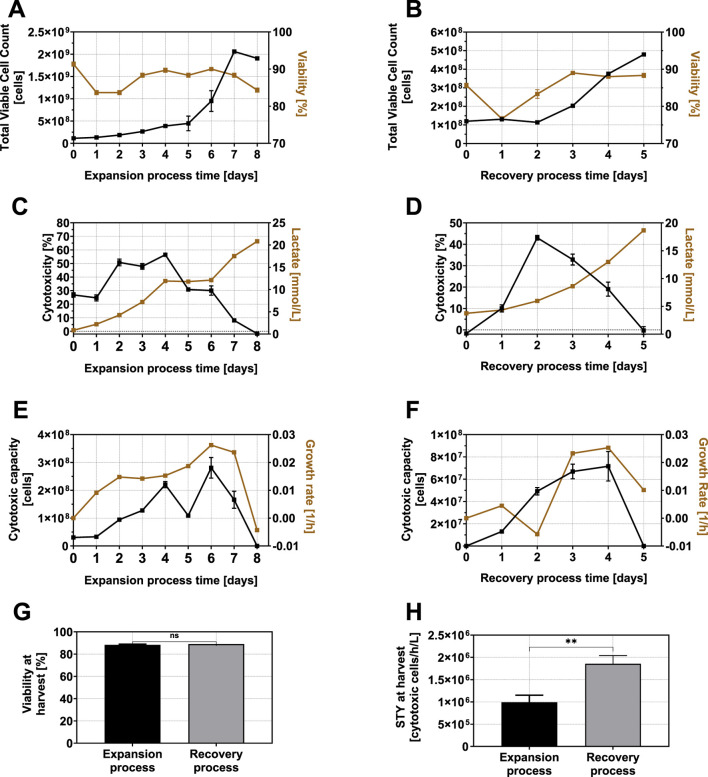
Total viable cell count and viability **(A)**, cytotoxicity and lactate concentration **(C)**, and cytotoxic capacity and specific growth rate **(E)** of NK-92 cells during a fed-batch expansion process in a 2-L working volume stirred-tank bioreactor. Total VCC and viability **(B)**, cytotoxicity and lactate concentration **(D)**, and cytotoxic capacity and specific growth rate **(F)** of NK-92 cells during the cytotoxicity recovery phase following expansion in the same bioreactor are shown. Due to the limited working volume of the bioreactor, only 6.6% of all cells produced during the fed-batch phase could be retained for the recovery phase (hence, different absolute values of the total viable cell count and cytotoxic capacity are displayed). Viablity at harvest **(G)** and STY at harvest **(H)** after the expansion process phase and the recovery process phase were compared uising an unpaired t-test where p ≥ 0.05 is not significant (ns), p < 0.05 is *, p < 0.01 is **, p < 0.001 is ***, and p < 0.0001 is ****. Error bars for expansion process and recovery process represent technical triplicates (n = 3).

To verify the feasibility of a two-phased expansion and subsequent cytotoxicity recovery strategy under controlled and scalable conditions, a fed-batch NK-92 expansion was conducted in a 2-L STR. During the expansion phase, NK-92 cells grew exponentially and reached a maximum viable cell count of 2.02 × 10^9^ ± 9.02 × 10^7^ cells by day 8, with a peak specific growth rate of 0.026 h^-1^ (Figure 4A), which was comparable with the flask-based observations. Viability remained high throughout most of the process, with a minor decrease after inoculation and towards the end of cultivation (Figure 4A).

Cytotoxicity followed a distinct temporal pattern: it peaked on day 4 at 56.4% ± 0.5% and subsequently declined to undetectable levels by day 8 (Figure 4C). These kinetics closely mirrored those observed in the small-scale experiments, although the maximum cytotoxicity reached in the STR was approximately 13% higher, potentially due to improved mixing and oxygen supply.

To assess the influence of lactate, a fed-batch phase from day 4 to 6 was performed to maintain lactate levels at a stable concentration (11.9 ± 0.1 mM throughout the fed-batch phase) (Figure 4C). Despite this control, cytotoxicity decreased to 30.4% ± 2.0% during this period, confirming the inhibitory effect of lactate rather than a decreased pH, as previously inferred from correlation analyses. Cytotoxic capacity increased in parallel with cell expansion until day 4, dropped temporarily, then rose again due to the increase in cell numbers at the end of the fed-batch phase, before ultimately falling below detection levels by day 8 (Figure 4E).

These results confirmed that cytotoxicity loss occurred during expansion even under pH and lactate-controlled conditions, highlighting the need for a dedicated cytotoxicity recovery phase to restore the effector function.

#### 3.3.2 Cytotoxicity recovery under optimized conditions in the STR

Following expansion, a recovery phase was implemented directly in the same bioreactor using the previously predicted optimal conditions (90% fresh cultivation medium and 37.2 °C incubation). A brief adaptation phase due to a re-inoculation was observed during the first 2 days of the cytotoxicity recovery process, with slight decreases in viable cell count and viability (Figure 4B). From day 3 onward the viable cell count and viability remained steady or even increased. Cytotoxicity recovered rapidly, reaching a peak of 43.1% ± 8.2% on day 2 (approximately 23.6% below the peak during expansion), followed by a decline as lactate levels rose again toward 10 mM (Figure 4D). Cytotoxic capacity continued to rise until day 3, reaching 6.69 × 10^7^ ± 5.35 × 10^6^ cytotoxic cells, and remained at a high level until day 4 before dropping sharply (Figure 4F). While the viability at harvest was comparable between the expansion and recovery phases (Figure 4G), the STY was significantly higher following the recovery process, highlighting its great advantage for improved cell function (Figure 2H).

## 4 Discussion

### 4.1 Recovery of cytotoxicity after NK-92 cell expansion

This study demonstrates the possibility and feasibility of recovering NK-92 cell cytotoxicity and cytotoxic capacity rapidly and efficiently by bioprocessing means only. Our findings highlight that the cultivation medium composition - particularly the proportion of fresh cultivation medium - is the primary driver of successful NK-92 cell cytotoxicity restoration. This also aligns with available literature, which emphasizes the necessity of cultivation medium replenishment to mitigate metabolic inhibition (Min et al., 2018; Granzin et al., 2015; Sutlu et al., 2010). Notably, the recovery duration exhibited a clear non-linear relationship with cytotoxic outcomes, peaking around day 3 before declining. This transient peak underscores the importance of precise harvest timing to maximize the cytotoxicity of cultivated cells. Unlike previous findings that highlighted temperature and oxygen as critical factors (von Werz et al., 2024), our analysis revealed only a minor impact of temperature and no significant effect from varying oxygen levels under standard cultivation conditions. This suggests that oxygen availability at typical cultivation concentrations meets the NK-92 metabolic demands during a short-term recovery. However, because hypoxia represents a hallmark of the TME (Wang et al., 2023; Du et al., 2021; Zhou et al., 2023), it represents an interesting topic for further investigation. In this study, these observations enabled a dimensionality reduction for analytical purposes, whereby variations in oxygen and temperature were pooled under the dominant factor of cultivation medium composition to facilitate clearer two-dimensional data visualization. This approach was chosen to simplify interpretability, as medium composition emerged as the primary driver of cytotoxicity recovery. Importantly, the data remain largely presented in a time-resolved manner, and not all experimental dimensions were collapsed. Nevertheless, although temperature was not identified as a primary determinant, it did contribute to cytotoxic outcomes. We therefore acknowledge that pooling across conditions differing in additional DoE factors may affect statistical independence and potentially overestimate precision, representing a limitation of this analytical simplification.

Due to a highly susceptible nature of K562 cells to NK-92 cell-mediated lysis, we determined the cytotoxic potential of the cultivated NK-92 cells using this approach by employing flow cytometry, the method which is also considered an equivalent to the “gold standard” K562 killing assays using radioactive isotopes (Chrobok et al., 2019; Penney McWilliams-Koeppen, 2022; Liu et al., 2002; Langhans et al., 2005). This killing assay is well established, documented and allows comparison of cytotoxic activity across different studies. It was proved that the E:T ratio of 1:1 is suitable for the assay performance, which (compared to a 24 readout) had a higher sensitivity after 4 h co-culture incubation time (Langhans et al., 2005), as used also in our study. Additionally, in the last decades numerous studies performed with NK cells successfully used this approach to determine the cytotoxicity of NK cells (Bazhenov et al., 2020; Xu et al., 2025; Saito et al., 2013; Eskandarian et al., 2026). The reason for the frequent use of K562 cells is their robustness in the killing assay their low viability is due to effector killing rather than poor culturing conditions. However, the results of this killing assay provided us with a primary information about the NK-92 behavior and some further experiments on additional tumor target cell lines (e.g., Raji, Rs4.11 or Jurkat cells) and ultimately animal models should be done as well to give a deeper insight of the NK-92 cytotoxicity performance, especially in clinical context.

With the aim to identify the possible molecular mechanisms underlying the differences observed between the cytotoxicity recovery in fresh, mixed, or spent cultivation medium, we performed extensive surface marker analysis. Interestingly, apart from modest differences in two markers (CD15s and CD183), no significant changes were detected in markers associated with activation, exhaustion, homing or chemokine reception. CD15s, a carbohydrate epitope involved in cell–cell adhesion, has been linked to NK cell trafficking and target recognition (Tang et al., 2025), while CD183 (CXCR3), a chemokine receptor, promotes chemokine-driven migration and has been associated with enhanced NK cell recruitment and a cytotoxic function (Groom and Luster, 2011; Inngjerdingen et al., 2001; Robertson, 2002). These findings suggest that the pronounced functional differences between recovery conditions were not attributable to surface marker expression, but rather to an intracellular mechanism obstructing cytotoxicity. It should be noted, however, that these speculations are only based on correlative observations and would require further experimental verification.

A key novel insight of this research work is the decoupling of cytotoxicity recovery from cell proliferation, which is in contrast to previous findings, linking improved function with renewed growth (Stenger and Miller, 2024). Modest proliferation was observed exclusively in mixed cultivation medium, while fresh cultivation medium promoted higher cytotoxicity without significant cell growth. This finding diverges from a conventional understanding (Stenger and Miller, 2024) and emphasizes distinct metabolic requirements for improved cytotoxic functionality *versus* proliferation.

Within the complex networks of NK cell immunometabolism regulation (Husain et al., 2013; Sohn and Cooper, 2023; O'Brien and Finlay, 2019; Poznanski et al., 2021) lactic acid and/or lactate proved to inhibit the cytolytic function of NK cells (Jin et al., 2025; Jedlicka et al., 2022; Brand et al., 2016; Husain et al., 2013; Har et al., 2019). Strong negative correlations between initial lactate levels and cytotoxicity recovery underline the importance of metabolite management (i.e., medium exchange), which is consistent with established cell cultivation practices (Huang et al., 2023; Quinn et al., 2020). However, our results uniquely demonstrate that lactate reduction and pH normalization are crucial, specifically for cytotoxic function restoration, not merely growth support. It should be noted, however, that these speculations are only based on correlative observations and would require further experimental verification.

Predictive PLS modeling identified an optimal recovery scenario of 3.7 days, 90% fresh cultivation medium and a slightly elevated temperature (37.2 °C). These conditions strategically support maximum cytotoxicity and we further believe that they could be also beneficial in means of cost and process efficiency, even with only a partial rather than complete medium replacement.

Overall, our study proposes an innovative two-phase cultivation approach, separating expansion from cell cytotoxicity recovery. This novel cultivation strategy could simplify the process control while increasing the NK-92 cytotoxicity at harvest and could therefore be used in future manufacturing approaches. This contrasts with typical single-phase processes, which attempt simultaneous optimization of growth and functionality, and could have a beneficial outcome on cost-effective and reliable NK-92 cell manufacturing.

### 4.2 Alignment with model predictions and scale-up considerations

The successful translation of the cytotoxicity recovery strategy from static small-scale experiments to a controlled STR bioreactor further validates its reproducibility and scalability. The STR expansion phase yielded exponential cell growth comparable to static cultivation performance, with notably higher peak cytotoxicity, likely attributed to the well-controlled pH, improved nutrient supply and optimized aeration. Despite lactate level control during fed-batch expansion, cytotoxicity declined, reinforcing the hypothesis of a specific lactate threshold of around 10 mM for uncompromised cytotoxicity. Similar levels of lactate were also previously reported without significantly decreased cytotoxicity (Huang et al., 2023). Exceeding this threshold repeatedly demonstrated loss of cytotoxicity in our analyses, highlighting the necessity of a dedicated cytotoxicity recovery phase in such instances. Cytotoxicity recovery in the STR, with the identified optimal conditions (90% fresh cultivation medium, 37.2 °C), aligned closely with predictions from the PLS model, with cytotoxicity and total cytotoxic cells reaching their peak within the expected 3- to 4-day window. While only a fraction of cells (i.e. 6.6%) could be transitioned to the cytotoxicity recovery bioreactor due to vessel size limitations, the process remains inherently scalable. At manufacturing scale, without vessel size limitations, the cytotoxic capacity could increase approximately 15-fold (as only 6.6% of the cells were transferred to the recovery bioreactor) compared to our current observations, given no additional scaling limitations occur. The sustained cytotoxic capacity achieved in the STR environment underscores the improved environmental control as a distinct advantage over static conditions. These results confirm the potential scalability and industrial applicability of our two-phase cultivation strategy, clearly distinguishing it from traditional single-phase or perfusion-based methods. This two-phased process could be implemented into existing production facilities without bioreactor modifications and would likely require much less cultivation medium than perfusion systems—potentially offering significant cost savings, while reducing the total manufacturing time per dose by several days (Granzin et al., 2015; Tam et al., 2003; Lapteva et al., 2012).

## 5 Conclusion

This study presents a novel framework for addressing cytotoxicity loss during NK-92 cell expansion and offers practical solutions for cytotoxicity recovery. Using a time-resolved DoE approach, we identified lactate as a critical factor influencing NK-92 cytotoxic function. Importantly, cytotoxicity recovery was decoupled from cell proliferation, supporting a two-phase cultivation strategy to optimize expansion and recovery separately. A frequently reported clinical dose of 2 × 10^9^ NK-92 cells was produced within a 7-day expansion phase, with a rapid cytotoxicity recovery within 3.7 days. Implementing this approach in a stirred-tank bioreactor demonstrated its scalability potential, indicating possible future suitability for improving cell manufacturing and consequently potentially improving the access to the patients.

## Data Availability

The original contributions presented in the study are included in the article/Supplementary Material, further inquiries can be directed to the corresponding authors.
