# Supplementary material for: A novel cultivation strategy to recover NK cell cytotoxicity
Source: Front Bioeng Biotechnol. 2026 Apr 2;14:1797129. doi: 10.3389/fbioe.2026.1797129 (PMC13083201; doi:10.3389/fbioe.2026.1797129)
Supplement: Supplementary file 1 [file Supplementaryfile1.pdf]

Supplementary material

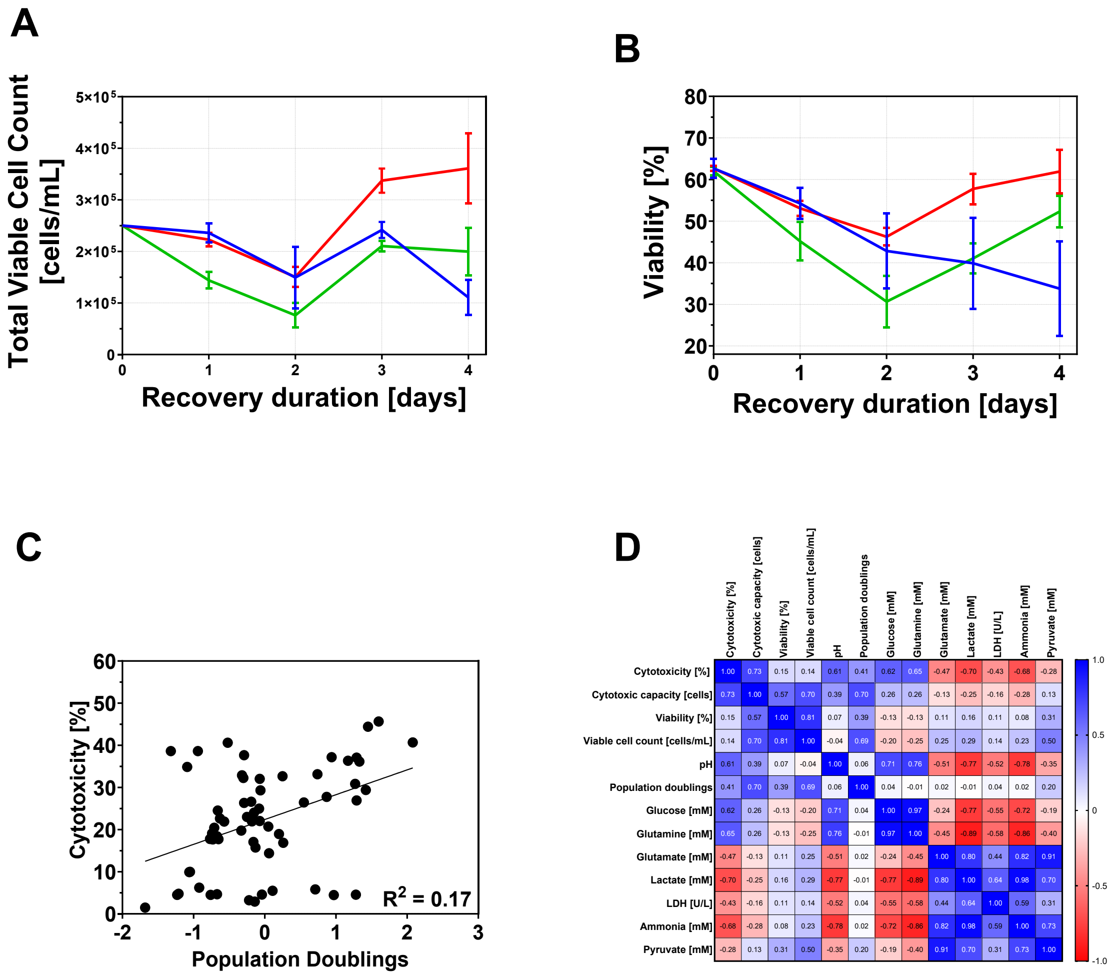

Supplementary figure 1 A) Time-resolved measurements of total viable cell count B) and viability during the recovery process. C) Linear regression between the cytotoxicity and the population doublings within the recovery phase. D) Pearson correlation matrix between all analyzed cultivation parameters.

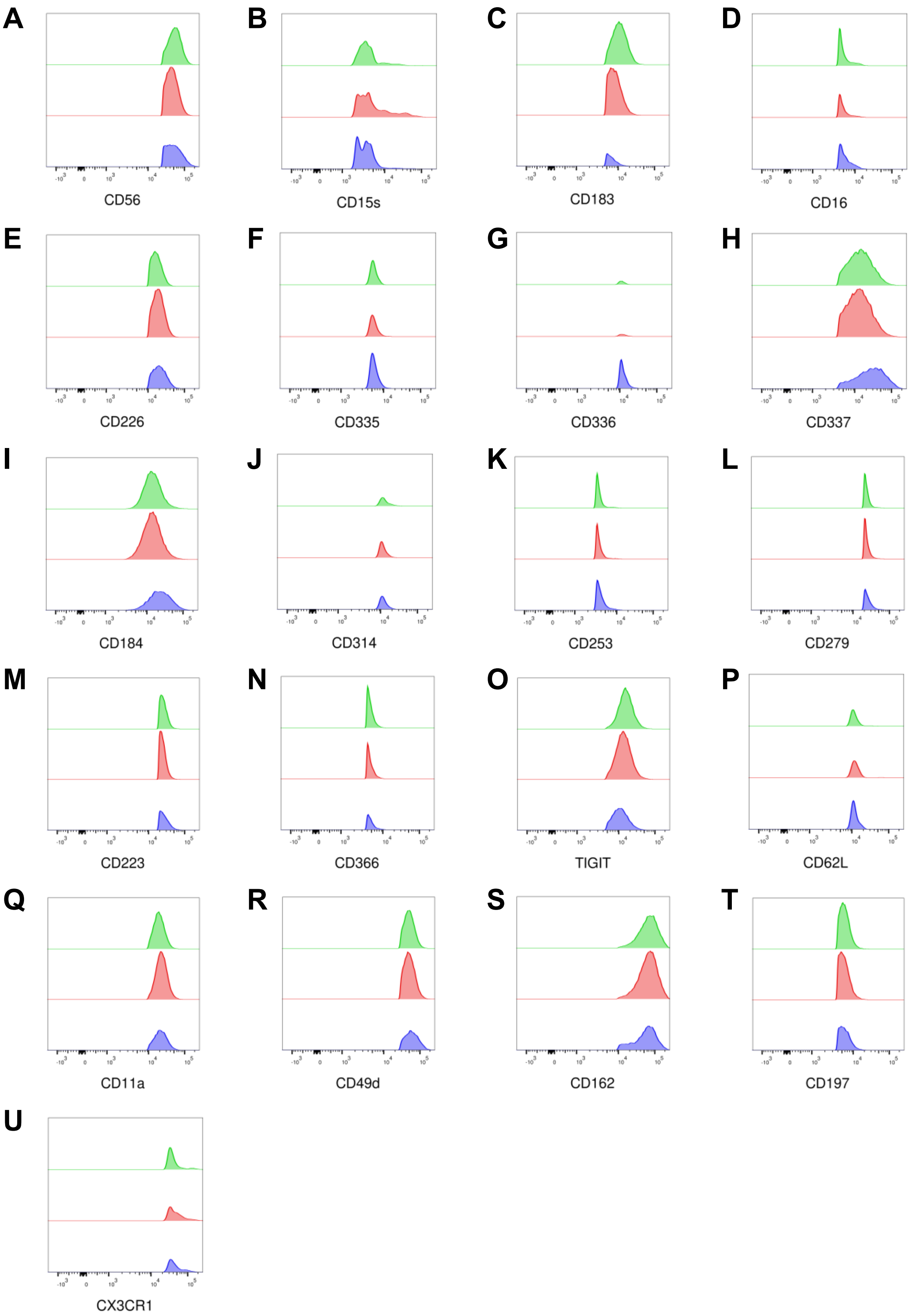

Supplementary figure 2 Stacked histograms of analyzed surface markers concatenated into groups of cultivation medium type (green represents fresh, red represents mixed and blue represents spent cultivation medium) on day 3 of the cytotoxicity recovery process. Shown is the mean flourescence intensity indicating the expression level of each surface marker in the resprective medium.

Supplementary table 1 Overview of factors and levels of the design space of the DoE.

Exp No.	Exp Name	Percent fresh medium	Atmospheric oxygen level [%]	Temperature [°C]	Time [days]
1	N1	0	10	37	1
2	N2	50	10	37	1
3	N3	100	10	37	1
4	N4	0	21	37	1
5	N5	50	21	37	1
6	N6	100	21	37	1
7	N7	0	10	39	1
8	N8	50	10	39	1
9	N9	100	10	39	1
10	N10	0	21	39	1
11	N11	50	21	39	1
12	N12	100	21	39	1
13	N13	0	10	37	2
14	N14	50	10	37	2
15	N15	100	10	37	2
16	N16	0	21	37	2
17	N17	50	21	37	2
18	N18	100	21	37	2
19	N19	0	10	39	2
20	N20	50	10	39	2
21	N21	100	10	39	2
22	N22	0	21	39	2
23	N23	50	21	39	2
24	N24	100	21	39	2
25	N25	0	10	37	3
26	N26	50	10	37	3
27	N27	100	10	37	3
28	N28	0	21	37	3
29	N29	50	21	37	3
30	N30	100	21	37	3
31	N31	0	10	39	3
32	N32	50	10	39	3
33	N33	100	10	39	3
34	N34	0	21	39	3
35	N35	50	21	39	3
36	N36	100	21	39	3
37	N37	0	10	37	4
38	N38	50	10	37	4
39	N39	100	10	37	4
40	N40	0	21	37	4
41	N41	50	21	37	4

42	N42	100	21	37	4
43	N43	0	10	39	4
44	N44	50	10	39	4
45	N45	100	10	39	4
46	N46	0	21	39	4
47	N47	50	21	39	4
48	N48	100	21	39	4
49	N49	50	10	39	1
50	N50	50	10	39	1
51	N51	50	10	39	2
52	N52	50	10	39	2
53	N53	50	10	39	3
54	N54	50	10	39	3
55	N55	50	10	39	4
56	N56	50	10	39	4
57	N57	50	21	37	1
58	N58	50	21	37	1
59	N59	50	21	37	2
60	N60	50	21	37	2
61	N61	50	21	37	3
62	N62	50	21	37	3
63	N63	50	21	37	4
64	N64	50	21	37	4

Supplementary table 2 Overview of all used antibodies, seperated into two staining panels, with the flourophore, volume per test, clone, and manufacturer.

Panel ID	Antibody target	Fluorophore	Volume per test [µl]	Clone	Supplier
PANEL 1	CD3	BUV395	2	SK7	BD Horizon
PANEL 1	CD14	FITC	10	M5E2	BD Pharmingen
PANEL 1	CD45	BUV805	1	HI30	BD Horizon
PANEL 1	CD56	PE	1	B159	BD Pharmingen
PANEL 1	CD16	APC	1	3G8	BD Pharmingen
PANEL 1	CD19	BV786	2	SJ25C1	BD Horizon
PANEL 1	CD62L	BUV496	5	DREG56	BD OptiBuild
PANEL 1	CD226	BUV737	1	11A8	BD OptiBuild
PANEL 1	CD335	BV510	3	9E2	BD Horizon
PANEL 1	CD162	BV650	2	KPL-1	BD OptiBuild
PANEL 1	CD336	RB780	2	P44-8	BD Horizon
PANEL 1	CD337	R718	1	P30-15	BD Horizon
PANEL 1	CD253	BV421	3	RIK-2	BD Horizon
PANEL 1	CD314	PerCP-Cy5.5	3	1D11	BD Pharmingen
PANEL 1	CD279	BV711	3	MIH4	BD OptiBuild
PANEL 1	CD223	BV605	3	T47-530	BD Horizon
PANEL 2	CD3	BUV395	2	SK7	BD Horizon
PANEL 2	CD14	FITC	10	M5E2	BD Pharmingen
PANEL 2	CD45	BUV805	1	HI30	BD Horizon

PANEL 2	CD56	PE	1	B159	BD Pharmingen
PANEL 2	CD16	APC	1	3G8	BD Pharmingen
PANEL 2	CD19	BV786	2	SJ25C1	BD Horizon
PANEL 2	CD11a	BUV496	2	HI111	BD OptiBuild
PANEL 2	CD49d	BUV615	1	9F10	BD Horizon
PANEL 2	CX_3CR1	BUV737	2	2A9-1	BD OptiBuild
PANEL 2	CD184	BV650	2	12G5	BD OptiBuild
PANEL 2	TIGIT	RB780	2	TgMab-2	BD Horizon
PANEL 2	CD183	R718	5	1C6/CXCR3	BD Horizon
PANEL 2	CD15s	BV421	2	CSLEX1	BD Horizon
PANEL 2	CD366	PerCP-Cy5.5	3	7D3	BD Pharmingen
PANEL 2	CD197	BV711	3	2-L1-A	BD Horizon

Supplementary table 3 List of components/latent variables used to generate the models for cytotoxicity and cytotoxic capacity

		**Component 1**
**Cytotoxicity**	R2	0.841653
	Q2	0.787348
N = 64	Cond. no. =	4.051
Components = 1		
**Cytotoxic capacity**	R2	0.62382
	R2adj	0.598316
	Q2	0.600918
N = 64	Cond. no. =	2.6
Components = 1		
